# Investigating the Quantitative Structure-Activity Relationships for Antibody Recognition of Two Immunoassays for Polycyclic Aromatic Hydrocarbons by Multiple Regression Methods

**DOI:** 10.3390/s120709363

**Published:** 2012-07-09

**Authors:** Yan-Feng Zhang, Li Zhang, Zhi-Xian Gao, Shu-Gui Dai

**Affiliations:** 1 Key Laboratory for Pollution Process and Environmental Criteria of Ministry of Education, College of Environmental Science and Engineering, Nankai University, Tianjin 300071, China; E-Mails: zhangli@nankai.edu.cn (L.Z.); sgdai@nankai.edu.cn (S.-G.D.); 2 Institute of Hygiene and Environmental Medicine, Tianjin 300050, China; E-Mail: gaozhx@163.com

**Keywords:** polycyclic aromatic hydrocarbons, immunoassay, enzyme-linked immunosorbent assay, cross-reactivity, quantitative structure-activity relationship, hapten

## Abstract

Polycyclic aromatic hydrocarbons (PAHs) are ubiquitous contaminants found in the environment. Immunoassays represent useful analytical methods to complement traditional analytical procedures for PAHs. Cross-reactivity (CR) is a very useful character to evaluate the extent of cross-reaction of a cross-reactant in immunoreactions and immunoassays. The quantitative relationships between the molecular properties and the CR of PAHs were established by stepwise multiple linear regression, principal component regression and partial least square regression, using the data of two commercial enzyme-linked immunosorbent assay (ELISA) kits. The objective is to find the most important molecular properties that affect the CR, and predict the CR by multiple regression methods. The results show that the physicochemical, electronic and topological properties of the PAH molecules have an integrated effect on the CR properties for the two ELISAs, among which molar solubility (*S*_m_) and valence molecular connectivity index (^3^χ^v^) are the most important factors. The obtained regression equations for Ris^C^ kit are all statistically significant (*p* < 0.005) and show satisfactory ability for predicting CR values, while equations for RaPID kit are all not significant (*p* > 0.05) and not suitable for predicting. It is probably because that the Ris^C^ immunoassay employs a monoclonal antibody, while the RaPID kit is based on polyclonal antibody. Considering the important effect of solubility on the CR values, cross-reaction potential (CRP) is calculated and used as a complement of CR for evaluation of cross-reactions in immunoassays. Only the compounds with both high CR and high CRP can cause intense cross-reactions in immunoassays.

## 1. Introduction

Polycyclic aromatic hydrocarbons (PAHs) are ubiquitous contaminants found in air, water, sediment and soil. They are a large and diverse class of compounds consisting of two or more fused aromatic rings produced by both natural and anthropogenic processes. Since many PAHs and the metabolites are mutagens and carcinogens, PAHs have been listed as priority pollutants in many countries. PAHs rarely occur as individual compounds in the environment, but always as a complex mixture of various compounds. Conventional analytical methods for PAHs are gas chromatography (GC) and high-performance liquid chromatography (HPLC) which require time-consuming pretreatment extraction procedures.

In recent years, immunoassays have represented a fast, sensitive, inexpensive and field-portable analytical method to complement traditional chromatographic procedures for PAHs [1]. Some immunoassay techniques, such as enzyme-linked immunosorbent assay (ELISA) [2,3], fluorescence polarization immunoassay [4], chemiluminescent immunoassay [5], real-time immuno-polymerase chain reaction assay [6] and immunosensor [7], were developed for PAHs analysis. Several ELISA test kits for PAHs have been produced and are commercially available, among which RaPID and Ris^C^ are the most widely studied and used [8]. They have been used for determination of PAHs in water [9,10], soil [11–15], biological [16,17] and some other samples [18]. The Ris^C^ kit is used in US EPA method 4035 for rapid screening test of PAHs in soil samples.

Cross-reactions are common phenomena in immunoassays, *i.e.*, the antibody responds with compounds structurally related to the analyte. Cross-reactivity (CR) is an essential character to evaluate the extent of cross-reactions in immunoreactions and immunoassays. Although CR affects the specificity of the immunoassay and possibly results in bias in the test result, sometimes it can be explored to identify metabolites or structurally similar compounds of the analyte for class-specific immunoassays. It is believed that the CR of a cross-reactant is determined by the physicochemical and structural properties of the compound.

In our previous work, we found that CR values of PAHs are significantly correlated with the data of logarithm of octanol-water partition coefficient (log *K*_ow_), but it seemed that CR cannot be solely explained by log *K*_ow_ [19]. In this article, in addition to log *K*_ow_, other six representative physicochemical, electronic and topological descriptors are selected to investigate the correlation between CR and the molecular properties of PAHs. Three multiple regression methods, including stepwise multiple linear regression (MLR), principal component regression (PCR) and partial least square regression (PLSR), were employed to develop the quantitative structure-activity relationship (QSAR) models using the data of two commercial PAHs ELISA kits. The objective of this work is to find the most important molecular properties that affect the CR of PAHs in immunoassays, and if possible, to find the feasibility of predicting CR by multiple regression analysis. Moreover, considering the important effect of water solubility on the CR, we calculated cross-reaction potential (CRP) and used it to complement CR for evaluation of cross-reactions in immunoassays.

## 2. Experimental Section

### 2.1. Molecular Descriptor Data Set

**Figure 1.**
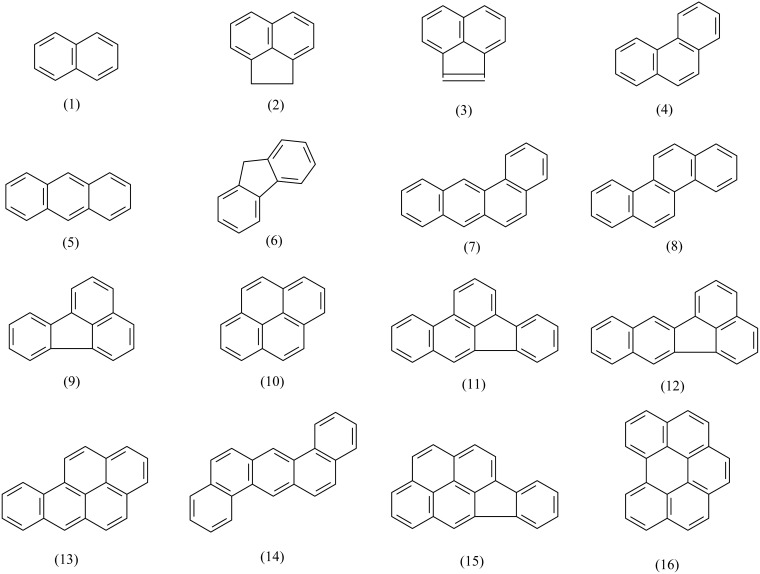
Molecular structures of the 16 studied PAHs.

The data of 16 representative PAHs (Figure 1) analyzed by the two ELISA kits were used for modeling. Seven typical physicochemical, electronic and topological descriptors are selected for developing the QSAR models. They are molar solubility (*S*_m_), the logarithm of octanol-water partition coefficient (log *K*_ow_), the gap between the highest occupied molecular orbital energy and the lowest unoccupied molecular orbital energy (*E*_HOMO_ − *E*_LUMO_), and four valence molecular connectivity indices (^0^χ^v^, ^1^χ^v^, ^2^χ^v^, ^3^χ^v^). The data of solubility (*S*) and log *K*_ow_ were obtained from Mackay *et al.* [20]. *S*_m_ was calculated by dividing *S* with the molecular weight. The data of *E*_HOMO_ − *E*_LUMO_ were from de Lima Ribeiro and Ferreira [21]. The data of ^0^χ^v^, ^1^χ^v^, ^2^χ^v^ and ^3^χ^v^ were cited from Govers and Aiking [22].

### 2.2. Data Set of Cross-Reactivity

The experimental data of cross-reactivity (CR) for the two commercial PAHs ELISA kits, RaPID and Ris^C^, were obtained from Krämer [8]. Since activity data used for QSARs should be in molar dimensions, CR values were converted to molar cross-reactivity (MCR), *i.e.*, the ratio of the molar IC_50_ of target analyte and the cross-reactant, for QSAR modeling [19]. Then, the predicted MCR was calculated by the QSAR models and converted to CR for comparison with the experimental CR value. We assumed that the CR values “<0.5%” and “<1.6%” were low enough to describe low levels of cross-reactions in the two ELISA kit tests, and reasonably considered the CR value “<” to be “=” for regression modeling [19].

### 2.3. Regression Analysis

The data of 14 compounds of the 16 PAHs were submitted as training set for regression analysis, and anthracene and benzo[a]pyrene were used as the test set. In order to reduce the colinearity and the number of the molecular descriptors, the analysis of the quantitative relationship between log MCR and the molecular descriptors was performed by stepwise MLR, PCR and PLSR employing SAS 8.1 software. In the stepwise MLR procedure, the data of the seven descriptors of the 16 PAHs were collected in a single data matrix, and the key descriptors were selected by adding descriptors one by one to perform a multivariable regression calculation. The variables significant at the 0.15 level were left in the model. In PCR analysis, the original descriptors were subjected to principal component analysis, and the subset of principal components explaining more than 90% of the variance was extracted. Then, the principal components extracted were subjected to multiple linear regression analysis. The PLSR method reduced large volume of descriptors to several components that were most correlative with the CR. These components were the linear combinations of the descriptors and used as new variables for regression analysis. The optimum number of components for regression analysis was obtained by the leave-one-out cross-validation procedure.

### 2.4. Cross-Reaction Potential

CR is the ratio of the IC_50_ (the 50% inhibition concentration) of the target analyte and the IC_50_ of the cross-reactant. Considering the effect of water solubility on the CR value and the immunoassay results, we defined cross-reaction potential (or cross-reaction probability, CRP), *i.e.*, 100-fold the ratio between the solubility (*S*) of a cross-reactant and the IC_50_ value [Equation (1)], and used it as a complement of CR to evaluate the extent of cross-reaction. CRP reflects the relative extent of cross-reaction of a non-target cross-reactant compared with the water solubility. The data set of *S* was from Mackay *et al.* [20], and IC_50_ data were from Krämer [8]. We assumed that the IC_50_ value “>1,000 μg·L^−1^” was high enough to be considered as “=1,000 μg·L^−1^” for CRP calculation:

(1) $$\text{CRP}(\%)=\frac{S}{IC_{50}}\times 100$$

## 3. Results and Discussion

### 3.1. Effect of Molecular Properties on Cross-Reactivity

The molecular structures of the 16 PAHs analyzed by the two ELISAs are shown in Figure 1. Since antibodies and antigens in immunoreactions are not mass-equivalent but rather molar-equivalent, molar cross-reactivity (MCR) rather than mass cross-reactivity (CR) is applied to investigate the quantitative structure and cross-reactivity relationships. The obtained stepwise MLR, PCR and PLSR equations and statistical parameters are illustrated in Table 1.

It shows that the regression models for Ris^C^ kit are all significant (*p* < 0.005), while the models for RaPID kit are all not significant (*p* > 0.05). The probable reason is that Ris^C^ immunoassay employs a monoclonal antibody, while RaPID kit is based on polyclonal antibody. In the stepwise MLR procedure for RaPID, only *S*_m_ enters the regression model, and the other six molecular descriptors are excluded from the regression equation. As for Ris^C^, *S*_m_ and ^3^χ^v^ are left in the model. In the PCR procedure, the two most significant principal components (PC1 and PC2) describe respectively 85.7% and 8.3%, and totally 94.0% of the variance. Eigenvectors of the principal components indicate that PC1 demonstrates the integrated character of the seven descriptors, while PC2 mainly represents the character of *S*_m_. The regression equations for RaPID and Ris^C^ are Equation (2) and Equation (3) respectively. In the PLSR procedure, the models are optimized by leave-one-out cross-validation, and the optimum numbers of components are found to be 4 and 2 for RaPID and Ris^C^, respectively. The parameter estimates for centered and scaled data (marked by *) are shown in Equation (4) and Equation (5). The results of stepwise MLR, PCR and PLSR imply that *S*_m_ plays an important role in affecting the CR property of the PAHs for the two ELISA kits, and ^3^χ^v^ also affects the CR for Ris^C^ kit to some extent:

(2) For RaPID:log MCR=1.349+0.07442×PC1-0.7234×PC2

(3) $${\text{For Ris}}^{\text{C}}:\text{log MCR}=1.178+0.002550\times \text{PC}1-0.9075\times \text{PC}2$$

(4) For RaPID:logMCR∗=-0.6783×Sm∗+1.245×logKow∗-0.6106×(EHOMO-ELUMO)∗+0.2556×χ0v∗-0.07527×χ1v∗−0.6231×χ2v∗−1.626×χ3v∗

(5) For RisC:log MCR∗=-1.042×Sm∗-0.06846×logKow∗-0.1012×(EHOMO-ELUMO)∗-0.1649×χ0v∗-0.1778×χ1v∗-0.2025×χ2v∗-0.2517×χ3v∗

In immunoreactions and immunoassays, the interaction between antigen and antibody is caused by the complementary spatial distribution and the strong affinity between the antigen and the antibody, such as hydrogen bonds, electrostatic interactions, van der Waals forces and hydrophobic interactions. The strong effect of *S*_m_ on the CR properties of the two ELISAs reflects the important role of hydrophobic interactions in the PAH-antibody reactions, which confirms the previous result [19]. It is commonly believed that lower order molecular connectivity indices encode mainly the bulk of a molecule, whereas higher order indices encode more subtle features such as the presence of rings and branching patterns. The result that ^3^χ^v^ affects CR more than ^0^χ^v^, ^1^χ^v^ and ^2^χ^v^ implies that molecular shape is more influential than molecular size in the PAH-antibody reactions. It has been reported that *E*_HOMO_ and *E*_LUMO_ are responsible for the antibody recognition for phenylurea herbicides and organophosphorus pesticides [23–25]. These compounds consist of various functional groups and heteroatoms, while PAHs do not contain substituents and heteroatoms, hence electronic descriptors such as *E*_HOMO_ and *E*_LUMO_ may have minor effects on the antibody recognition for PAHs. *E*_HOMO_ − *E*_LUMO_ expresses the necessary energy to excite an electron from the highest occupied molecular orbital to the lowest unoccupied molecular orbital. Since immunoreactions are always not accompanied by a rearrangement of electron density, it is not surprising that *E*_HOMO_ − *E*_LUMO_ does not exhibit strong effect on the CR in the models.

### 3.2. Predicting Cross-Reactivity

**Figure 2.**
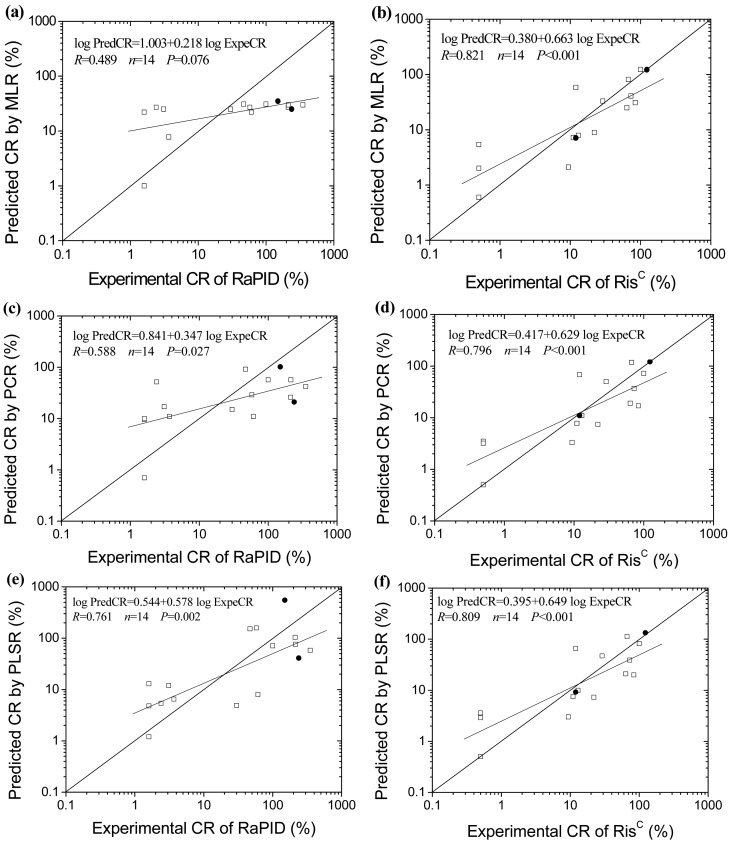
Plotting of predicted cross-reactivity (CR) *versus* experimental values of the PAHs ELISAs.

CR is one of the most important characteristics of an ELISA test, and influences the extent of cross-reaction and the results of ELISAs significantly. However, due to the difficulty and expense in term of cost and time, not all of the CR data of the cross-reactants are available. Moreover, it is impractical to directly measure the CR of the cross-reactants which are not commercially available, so predicted CR values of the PAHs for the two ELISA kits were calculated using the obtained MLR, PCR and PLSR models, and compared with the experimental data (Table 2, Figure 2). The predicted CR values for Ris^C^ agree very well with the experimental data, while the predicted and experimental data for RaPID do not agree well with each other. The models were further external validated using the data of three-ringed anthracene and five-ringed benzo[a]pyrene as test set. The range of predicting error for anthracene is from −1.6% to +8.1%, and for benzo[a]pyrene is from −40.8% to −8.3%. It appears that the obtained models can successfully predict the CR for Ris^C^ kit, but present poor predicting ability for RaPID kit.

### 3.3. Cross-Reaction Potential

Generally speaking, higher CR values imply higher levels of immunoreactions. However, the antigen-antibody reactions in immunoassays are carried out in water or buffer solutions, so if the solubility of a cross-reactant is much lower than the IC_50_ value, it cannot possibly cause intense cross-reactions in the immunoassays. That is to say, the concentration of this compound in real water samples cannot be high enough to evoke high extent cross-reaction in immunoassays, even though the CR is very high. Some of the 16 PAHs are very hydrophobic compounds, and the solubility is much lower than the tested IC_50_ value. For example, the IC_50_ referring to water analysis for benzo[a]pyrene for the RaPID ELISA kit is 6.9 μg·L^−1^, while the solubility of benzo[a]pyrene is 3.8 μg·L^−1^ (Table 3), so although benzo[a]pyrene has a high CR of 239% in the RaPID ELISA, the concentration of benzo[a]pyrene in water samples cannot be possibly high enough to evoke a high level of cross-reaction. Hence, considering the important effect of water solubility on immunoreactions and immunoassays, cross-reaction potential (or cross-reaction probability, CRP), *i.e.*, the relative IC_50_ of a non-target cross-reactant compared with its water solubility, was defined and used as a complement of CR to evaluate the potential and probability that a cross-reaction would occur.

**Figure 3.**
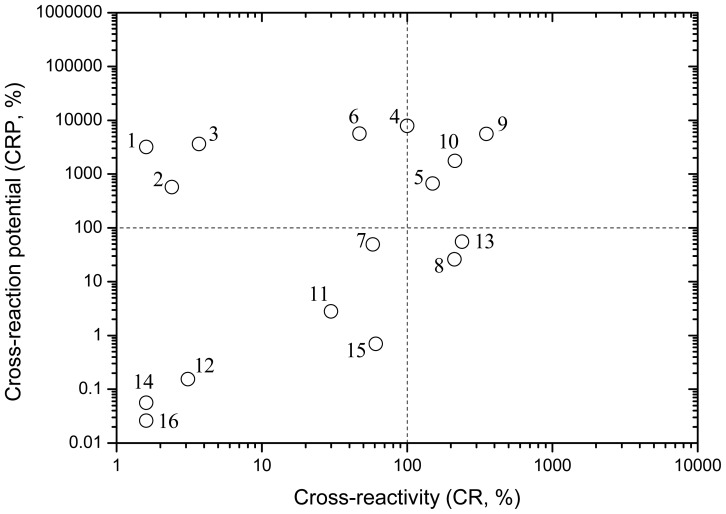
Cross-reaction potential (CRP) and cross-reactivity (CR) of 16 PAHs for RaPID ELISA kit.

The IC_50_ and CRP data for the 16 PAHs for RaPID kit are shown in Table 3. The CRP values for Ris^C^ kit are not calculated because the IC_50_ values are not available. In addition to the target analyte of phenanthrene, the 15 cross-reactants in RaPID ELISA can be divided into four groups according to CR and CRP (Figure 3): (I) CR > 100%, CRP > 100%; (II) CR > 100%, CRP < 100%; (III) CR < 100%, CRP > 100%; and (IV) CR < 100%, CRP < 100%. The compounds of group (I) might cause intense cross-reactions and affect the determination of phenanthrene, while the group (IV) compounds have little cross-reaction effect on the analysis results. As for the group (II) compounds, the CR is high, while the CPR is low because of the relatively low solubility. The group (III) compounds are two-ringed and three-ringed PAHs, and less cross-reactive but more water soluble. The extent of the cross-reactions of group (II) and (III) compounds depends on both the CR and the CRP properties.

It should be pointed out that the RaPID PAHs ELISA kit is applied not only for water samples [9,10], but more often for soil samples [11,14,15]. In the pretreatment procedure, PAHs were usually extracted from the soil samples by methanol and diluted by buffer. PAHs are very hydrophobic molecules and can be adsorbed to soils at very high concentration. In the immunoassays of PAHs, much attention should be paid to the solubility of the compounds during the procedures of solvent extraction and buffer dilution.

### 3.4. Comparison of the Two Kits

The comparison of RaPID and Ris^C^ PAHs ELISA kits based on the character and the applicability are illustrated in Table 4. It seems that Ris^C^ ELISA is more specific, while RaPID ELISA is applied for more kinds of environmental samples. The selection of appropriate ELISAs for PAHs depends on the objective and request of the analysis.

## 4. Conclusions

RaPID and Ris^C^ are two widely studied ELISA kits used for analysis of PAHs. Three regression methods, including stepwise MLR, PCR and PLSR, were successfully applied to investigate the correlation between the molecular properties and the CR properties of PAHs for the two ELISA kits. It seems that the physicochemical, electronic and topological properties of the PAH molecules have an integrated effect on the CR properties for the two kits. *S*_m_ and ^3^χ^v^ show especially strong effects on CR, which implies the important role of hydrophobic interactions and molecular shape in the PAH-antibody reactions. The obtained regression equations for Ris^C^ kit are all statistically significant (*p* < 0.005) and show satisfactory ability for predicting CR values, while equations for RaPID kit are all not significant (*p* > 0.05) and not suitable for prediction. It is probably because that the Ris^C^ immunoassay employs a monoclonal antibody, while the RaPID kit is based on polyclonal antibody. Considering the important effect of solubility on CR for the two PAHs ELISAs, cross-reaction potential (CRP) is defined and used as a complement of CR to evaluate the extent of cross-reaction in immunoassays. We believe that only the compounds with both high CR and high CRP can cause intense cross-reactions in immunoassays. This work demonstrated the feasibility of multiple regression methods in investigating the quantitative structure-CR relationships and predicting CR in immunoassays.

## Figures and Tables

**Table 1.** Regression models for quantitative structure and molar cross-reactivity (MCR) relationships for the PAHs ELISAs.

***ELISA kit***.	***Regression method***	***Regression Equation***	***Statistics***				

			***F***	***P***	***R****^2^*	***n***	**PRESS**
RaPID	MLR	log MCR = 1.542 − 0.00676 × *S*_m_	4.30	0.0603	0.2638	14	11.30
	PCR	log MCR = 4.907 − 0.01004 × *S*_m_ − 0.07337 × log *K*_ow_ − 0.2108 × (*E*_HOMO_ − *E*_LUMO_) − 0.04482 × ^0^χ^v^ − 0.06256 × ^1^χ^v^ − 0.06708 × χ^v^ − 0.4319 × ^3^χ^v^	3.17	0.0819	0.3656	14	16.68
	PLSR	log MCR = 10.49 − 0.008925 × *S*_m_ + 1.015 × log *K*_ow_ − 1.132 × (*E*_HOMO_ − *E*_LUMO_) + 0.1073 × ^0^χ^v^ − 0.04788 × ^1^χ^v^ − 0.4657 × ^2^χ^v^ − 8.925 × ^3^χ^v^	3.30	0.0634	0.5943	14	10.88
Ris^C^	MLR	log MCR = 3.439 − 0.01270 × *S*_m_ − 4.041 × ^3^χ^v^	12.19	0.0050	0.6603	14	4.62
	PCR	log MCR = 5.800 − 0.01231 × *S*_m_ − 0.1162 × log *K*_ow_ − 0.2169 × (*E*_HOMO_ − *E*_LUMO_) − 0.06868 × ^0^χ^v^ − 0.09743 × ^1^χ^v^ − 0.1065 × ^2^χ^v^ − 0.7045 × ^3^χ^v^	8.95	0.0049	0.6193	14	5.13
	PLSR	log MCR = 5.485 − 0.01245 × *S*_m_ − 0.05071 × log *K*_ow_ − 0.1704 × (*E*_HOMO_ − *E*_LUMO_) − 0.06290 × ^0^χ^v^ − 0.1027 × ^1^χ^v^ − 0.1375 × ^2^χ^v^ − 1.255 × ^3^χ^v^	9.71	0.0037	0.6383	14	4.92

**Table 2.** Experimental and predicted cross-reactivity (CR) of the PAHs ELISAs by regression analysis.

***Number***	***Compound***	***CR (*%*) of RaPID***				***CR (*%*) of Ris****^C^*			
	
		**Experimental** ^a^	**Predicted by MLR**	**Predicted by PCR**	**Predicted by PLSR**	**Experimental** ^a^	**Predicted by MLR**	**Predicted by PCR**	**Predicted by PLSR**
1	Naphthalene	<1.6	1.0	0.7	1.2	0.5	0.6	0.5	0.5
2	Acenaphthene	2.4	27	52	5.4	12	58	68	66
3	Acenaphthylene	3.7	7.8	11	6.5	13	7.9	11	10
4	Phenanthrene	100	31	57	71	100	123	72	82
5	Anthracene	150	35	102	550	123	122	121	133
6	Fluorene	47	31	92	152	67	81	118	113
7	Benzo[a]anthracene	58	27	29	158	64	25	19	21
8	Chrysene	212	27	26	104	84	31	17	20
9	Fluoranthene	351	30	42	58	73	41	37	39
10	Pyrene	214	30	57	76	29	33	50	47
11	Benzo[b]fluoranthene	30	25	15	4.9	22	8.9	7.4	7.3
12	Benzo[k]fluoranthene	3.1	25	17	12	11	7.2	7.8	7.5
13	Benzo[a]pyrene	239	25	21	41	12	7.1	11	9.2
14	Dibenzo[a,h]anthracene	<1.6	22	9.6	13	<0.5	5.4	3.5	3.6
15	Indeno[1,2,3-cd]pyrene	61	22	11	8.0	9.4	2.1	3.3	3.0
16	Benzo[g,h,i]perylene	<1.6	22	10	4.8	<0.5	2.0	3.2	2.9

a Data are from [8].

**Table 3.** Cross-reaction potential (CRP) of PAHs for RaPID ELISA kit.

***Number***	***Compound***	***S (mg*·*L*^−^***^1^*) ^a^	***IC****_50_* **(μ*g*·*L*^−^***^1^*) ^b^	***CRP (*%)**
1	Naphthalene	31.7	>1,000	3,170
2	Acenaphthene	3.93	688	571
3	Acenaphthylene	16.1	447	3,602
4	Phenanthrene	1.29	16.5	7,818
5	Anthracene	0.073	11	664
6	Fluorene	1.98	35.2	5,625
7	Benzo[a]anthracene	0.014	28.4	49
8	Chrysene	0.002	7.8	26
9	Fluoranthene	0.26	4.7	5,532
10	Pyrene	0.135	7.7	1,753
11	Benzo[b]fluoranthene	1.5 × 10^−3^	54.2	2.8
12	Benzo[k]fluoranthene	8.1 × 10^−4^	524	0.155
13	Benzo[a]pyrene	3.8 × 10^−3^	6.9	55
14	Dibenzo[a,h]anthracene	5.6 × 10^−4^	>1,000	0.056
15	Indeno[1,2,3-cd]pyrene	1.9 × 10^−4^	27.2	0.699
16	Benzo[g,h,i]perylene	2.6 × 10^−4^	>1,000	0.026

a Data are from [20];

b Data are referring to water analysis and from [8].

**Table 4.** Comparison of the Two PAHs ELISA kits.

	***RaPID***	***Ris****^C^*
Reference compound	Phenanthrene	Phenanthrene
Analysis mode	Competitive heterogeneous ELISA, antibody is coated on tubes	Competitive heterogeneous ELISA, antibody is immobilized to magnetic particles
Cross-reactivity	1.6%∼351%	0.5%∼123%
Specificity	Not so specific	Relatively specific
Antibody	Polyclonal	Monoclonal
Cross-reactant	Anthracene, chrysene, fluoranthene, pyrene and benzo[a]pyrene	Anthracene
Samples	Water, soil, biological and some other samples	Mostly soil samples till now
